# Mesoscopic Optical Imaging of the Pancreas—Revisiting Pancreatic Anatomy and Pathophysiology

**DOI:** 10.3389/fendo.2021.633063

**Published:** 2021-03-04

**Authors:** Tomas Alanentalo, Max Hahn, Stefanie M. A. Willekens, Ulf Ahlgren

**Affiliations:** Umeå Centre for Molecular Medicine, Umeå University, Umeå, Sweden

**Keywords:** mesoscopic imaging, optical projection tomography, light sheet fluorescence microscopy, pancreas, diabetes

## Abstract

The exocrine-endocrine multipart organization of the pancreas makes it an exceedingly challenging organ to analyze, quantitatively and spatially. Both in rodents and humans, estimates of the pancreatic cellular composition, including beta-cell mass, has been largely relying on the extrapolation of 2D stereological data originating from limited sample volumes. Alternatively, they have been obtained by low resolution non-invasive imaging techniques providing little detail regarding the anatomical organization of the pancreas and its cellular and/or molecular make up. In this mini-review, the state of the art and the future potential of currently existing and emerging high-resolution optical imaging techniques working in the mm-cm range with μm resolution, here referred to as mesoscopic imaging approaches, will be discussed regarding their contribution toward a better understanding of pancreatic anatomy both in normal conditions and in the diabetic setting. In particular, optical projection tomography (OPT) and light sheet fluorescence microscopy (LSFM) imaging of the pancreas and their associated tissue processing and computational analysis protocols will be discussed in the light of their current capabilities and future potential to obtain more detailed 3D-spatial, quantitative, and molecular information of the pancreas.

## Introduction

Both in rodents and humans, the compound organization of the pancreas renders analyses of its cellular and molecular make up exceedingly challenging. Until recently, studies of pancreatic anatomy/pathophysiology have largely relied on traditional immunohistochemical analyses. However, these usually only cover a very limited tissue volume and their results inevitably represent extrapolations of a limited amount of 2-dimensional (2D) data. This not only entails challenges for putting the resultant data into a 3-dimensional (3D) context, but it may also involve a wide range of assumptions regarding structural shapes (e.g., islets) or organ homogeneity of the object to study. Albeit stereological assessments obviously are of great importance; the development of modern optical imaging techniques provides a whole new set of tools to better assess and understand cellular and molecular pancreatic features in both a 3D spatial and quantitative context. By high resolution mesoscopic imaging techniques working in the mm-cm range, such as: optical projection tomography (OPT) (1) and light sheet fluorescence microscopy (LSFM) (2–4), highly detailed images of the pancreatic cellular composition can be obtained in a broader anatomical context, even within the framework of the entire gland. Furthermore, they offer powerful possibilities to assess various and specific quantitative and spatial pancreatic features in the diabetic setting, including ß-cell mass (BCM), distribution and plasticity, organ heterogeneities, rare event screening, cellular ratios, immune-cell infiltration, vascularization, innervation, and many more. When using advanced image analysis software, they also offer new ways to display the obtained data and perform advanced statistical assessments. In this mini-review, we will give a brief overview of how these techniques have been applied in basic diabetes research, elaborate on recent developments in the field and propose how they may be implemented in the future.

## OPT and LSFM: Complementary Approaches for *Ex Vivo* 3D Imaging of the Pancreas

Although 3D imaging techniques, such as: micro-computed tomography (µCT) and micro-magnetic resonance imaging (µMRI), provide high resolution images of tissue morphology in complete organs, or even entire animals, they cannot take advantage of the molecule-specific labeling techniques available for fluorescence microscopy imaging. OPT and LSFM are optical 3D imaging techniques for mesoscopic sized samples (i.e., on the mm-cm scale), enabling assessments on the mm-scale. In principle, OPT could simply be described as the optical analogue of X-ray CT, using light instead of X-rays. Hence, by acquiring a series of 2D projections, obtained from different angles, the 3D structure of the investigated tissue is generated as a stack of cross-sectional image slices using a reconstruction algorithm, commonly a filtered-back projection. In contrast to OPT, wherein sample illumination and detection are performed in one direction (for each projection), LSFM acquires data by performing these processes in two distinct optical paths, orthogonally to each other. Most commonly, a light sheet is generated by a (Gaussian) laser and the specimen is scanned either by moving the light sheet or by moving the specimen in relation to a static light sheet in order to produce optical sections through the specimen. For both techniques, registration is performed by a digital camera. Whereas OPT and LSFM have several common features, including labeling techniques and the need for tissue clearing, allowing light propagation through the tissue sample, they also have a number of distinct features, rendering both of them optimal for different imaging tasks. Most importantly, LSFM provides a higher lateral but lower axial resolution as compared to OPT (5), resulting in non-isotropic voxels, which may result in ambiguities for 3D analysis. Nonetheless, it should be noted that both hardware and software development is advancing rapidly in this field. A recent significant example is cleared-tissue axially swept light-sheet microscopy (ctASLM), which enables imaging with significantly increased z-axial resolution in mm-sized specimen (6), thereby approaching isotropic voxels. However, due to its respective detection principle, OPT does provide true isotropic spatial resolution (i.e., voxels with identical dimensions along the x, y, and z axes). For imaging of murine and human pancreatic tissue, it is our experience that OPT is more suited for larger samples at lower magnification and for larger tissue cohorts, in relation to scan times and data volumes, while LSFM is preferred for higher resolution images of specific regions of interest (ROIs), or smaller sample cohorts. Still, both techniques are often interchangeable and can in many cases substitute or complement each other (5).

Methods for rendering tissues transparent were already described as early as in 1914 (7) F and represent a key feature in mesoscopic 3D imaging to allow the propagation of light through the investigated specimen. Another very important aspect is to assure penetration of labeling agents, commonly antibodies. For certain tissues, quenching of autofluorescence (AF) is also required to increase the signal to noise ratio. Due to the limited available methods to overcome these obstacles, fluorescent immunohistochemistry has remained, for a long period of time, restricted to thin tissue sections or embryonic scale specimen. In 2007, Alanentalo et al., developed an approach enabling clearing and whole-mount immunohistochemistry for OPT-based assessments of the BCM distribution in the complete mouse pancreas (8). Afterward, the protocol was successfully applied to other organs, such as livers containing islet grafts (9). Ever since, there has been an explosion in this particular field and at present, numerous powerful clearing, and whole-mount immunohistochemistry protocols exist [for review see (10–12)]. Clearing methods are commonly divided into organic-, aqueous-, and hydrogel-based clearing techniques (13). These have in common that they use liquids to match the refractive index (RI) of the cellular constituents to reduce light scattering within the sample. Organic clearing methods usually dehydrate and delipidate the tissue before homogenization of the RI. The choice of the optimal clearing agent for a specific tissue should be based on the sample size, the need to prevent fluorescence quenching of the labeling reagent (like transgenically expressed proteins), and its potential tissue shrinkage or expansion effects.

## Mesoscopic Imaging of the Pancreas—So Far

**Figure 1:**
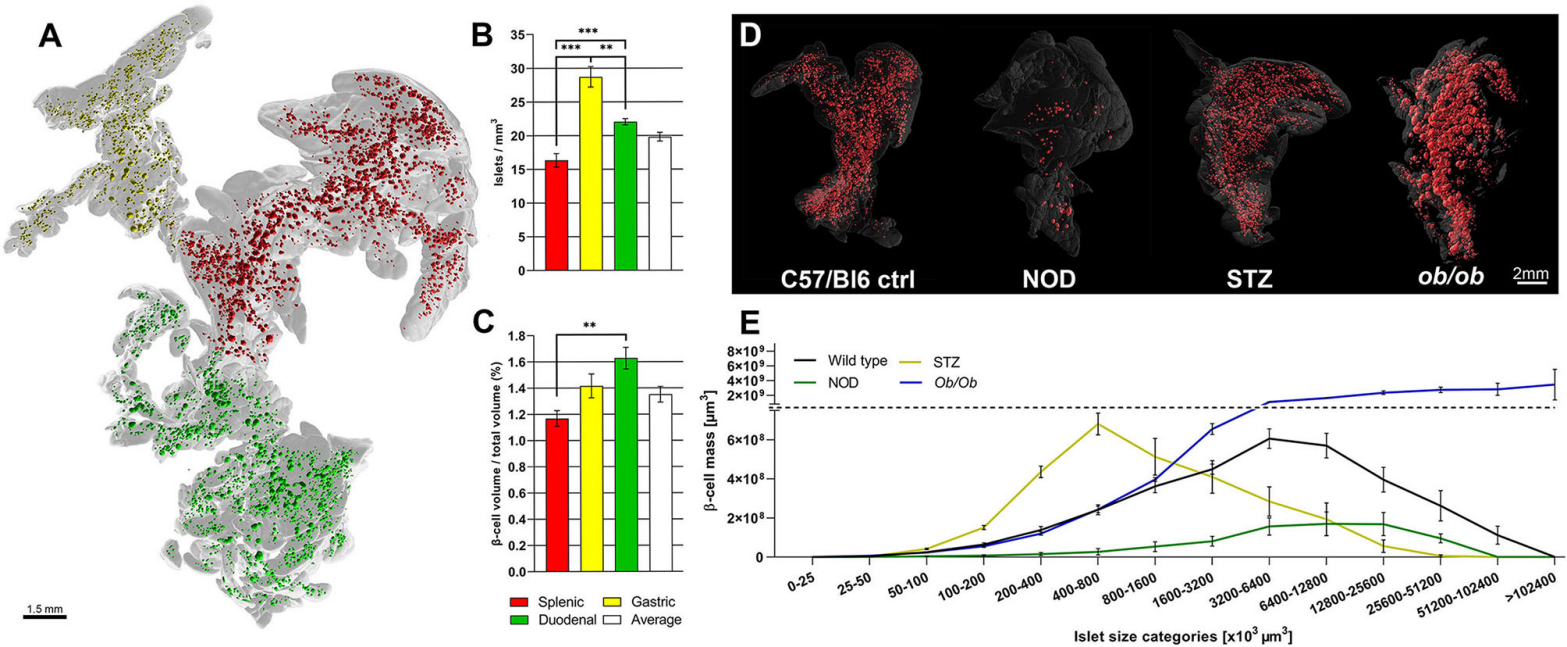
Optical projection tomography enables isotropic resolution imaging and quantification of antibody labeled features throughout the volume of the rodent pancreas. Montage illustrating the variance in islet mass distribution between different disease models as determined by OPT. **(A)** Optical projection tomography (OPT) generated 3D reconstruction of the isolated pancreas from a C57Bl/6 mouse (8 weeks) labeled for insulin. The islets of the splenic, gastric, and duodenal lobe are pseudocolored; red, yellow, and red respectively. The lobular compartments (gray) are delineated based on their developmental origins [see Hörnblad et al. (14)]. **(B, C)** Graphs showing the lobar distribution of insulin^+^ islets per mm (3) **(B)** and ß-cell volume per total lobular volume **(C)**, illustrating that the lobular compartments of the rodent pancreas display significant different differences in islet ß-cell mass (BCM) densities. **(D)** Images depicting (from left to right) OPT based iso-surface reconstructions of adult mouse pancreas (splenic lobe), from a healthy C57Bl/6 mouse (8 weeks), a non-obese diabetic (NOD) mouse (T1D model at 16 weeks), streptozotocin (STZ) induced diabetic (C57Bl/6 mice at 9 weeks, 2 weeks post-single high dose STZ administration), and an *ob/ob* mouse (at 26 weeks), respectively. The islet β-cell volumes (red) are reconstructed based on insulin specific antibody signal and the outline of the pancreas (gray) is based on the tissues autofluorescence. **(E)** Graph depicting ß-cell volumes per islet size category of the models depicted in **(D)**, illustrating the possibility to obtain detailed assessments of BCM distribution in the pancreas by OPT. Data is obtained from Hörnblad et al. (21), Alanentalo et al. (22), Parween et al. (23, 24), and Hahn et al. (25).

Pancreatic development has been well studied (14), but given the complex morphogenesis of the pancreatic buds in relation to one another and to the surrounding tissues, several aspects of its development are difficult to interpret by traditional 2D stereological imaging techniques. Therefore, OPT was originally used for 3D mapping of RNA and protein expression in developmental biology studies. The possibility of OPT to produce interactive 3D data, such as: blow-up views and tiltable section planes, highlighting distinct tissue components, has contributed to reveal new aspects of the interrelationship between the pancreatic epithelium and the spleno-pancreatic mesenchyme (15–17). Indeed, OPT imaging played an instrumental role in demonstrating that the gastric lobe of the murine pancreas forms by perpendicular growth from the dorsal pancreatic bud and, that its morphogenesis is dependent on formation of the spleen (18). Furthermore, these particular studies prompted for a designated nomenclature of the murine pancreatic regions, based on their developmental origins (14). After optimization of the OPT hardware, tissue clearing/labeling protocols and processing schemes for the murine pancreas, the complete BCM distribution within the intact gland, revealing significant heterogeneities in both islet size and number throughout the pancreatic lobes was presented (19).By further technical refinements (9, 20, 21), OPT has been proven a very useful approach for ß-cell/islet mass distribution assessments in murine diabetes models, by omitting the need for extrapolation of 2D data through direct assessments of the islet’s numbers, volumes, and spatial coordinates throughout the entire gland. Noteworthy, OPT analyses demonstrated significant heterogeneities in BCM distribution and islet density between the primary pancreatic lobes (21), emphasizing the need for careful considerations regarding the origin of the investigated tissue for sampled analyses (see **Figures 1A–C**). Working with tomographic data provides the possibility to perform combined 3D-spatial, quantitative, and statistical analyses of the BCM distribution (see **Figures 1D, E** **)**. As such, OPT has significantly contributed to a range of studies investigating BCM dynamics, islet plasticity, and ß-cell function in diverse rodent models (22, 23, 25–33). For example, by pseudocoloring islets of different size categories, patterns of selective islet vulnerability of different sized islets were revealed in models of ß-cell destruction. It was demonstrated that smaller islets are more susceptible to destruction in models of naturally induced diabetes (22, 28), whereas in streptozotocin induced diabetes primarily larger islets are affected (25). Furthermore, a global view of the pancreatic constitution may reveal novel features, even in previously well studied diabetes models. OPT analyses of the leptin deficient *Ob/Ob* mouse, a model used in thousands of investigations, demonstrated significant internal islet hemorrhaging, a feature overlooked in previous stereological assessments (23). Of note, in conjunction with this study, tomographic data of the complete pancreatic islet distribution between 4 and 52 weeks in cohorts of both lean and obese mice were made publicly available (24). These studies exemplify how OPT imaging of large animal cohorts can be employed to identify ROIs for higher resolution imaging by LSFM, in order to study disturbed islet morphology in more detail. Obviously mesoscopic imaging techniques like OPT and LSFM are not limited to studies of BCM. In particular LSFM techniques, taking advantage of its normally higher resolution, have been recently applied to study aspects of e.g., pancreatic innervation, immune cell infiltration, and proliferation, in various mouse models of diabetes using specific antibody markers for these features (34, 35).

*Ex vivo* OPT or LSFM imaging of the pancreas also provides a powerful complemental approach for studies of islet plasticity, destruction etc., conducted by *in vivo* assessments of islets grafts in the anterior chamber of the eye (ACE) (36) (see separate article Ilegems et al.). Since islets transplanted to this site have been reported to provide a mirror image of the endogenous islets, phenotypical *in vivo* observations in the ACE may therefore be confirmed on the pancreatic level by end point *ex vivo* mesoscopic imaging (23, 25, 37, 38). Additionally, mesoscopic optical imaging also offers the possibility to serve as an evaluating tool for other *in vivo* imaging technologies. Since they offer an “absolute” quantitative and spatial account of specifically labeled cells within the entire volume, OPT or LSFM data sets could serve as a “gold” standard against which other imaging modalities could be compared and potentially provide additional complementary information. In this respect, OPT has been demonstrated advantageous for validating radiotracer uptake for ß-cell imaging (39). Interestingly, this study demonstrated a superior linear correlation between the single photon emission computed tomography (SPECT) and OPT, as compared to SPECT *versus* histology. Another example comes from studies of islets with a technology diametrically different from SPECT. Ilegems et al., demonstrated that the transplanted islet volume to the ACE can be measured *in vivo* based on the backscatter from the insulin secretory granules. When the grafted eyes were examined by OPT *ex vivo*, the islet volumes could be plotted against each other, islet by islet, showing a nearly perfect correlation (40).

## Mesoscopic Imaging of the Pancreas—What Lies Ahead

In combination with a vast number of potent tissue-clearing protocols, mesoscopic optical imaging technologies have undergone dramatic developments during the past decade. We anticipate that the next, and very important, technological/methodological development for mesocopic imaging will involve improved procedures for penetration of fluorescent labeling reagents with high molecular binding specificity (e.g., antibodies). Such optimizations will help addressing one of the greatest limitations of these high-resolution 3D imaging approaches for diabetes research by allowing the possibility to analyze larger volumes of human pancreatic tissue. Albeit a small number of studies have demonstrated the possibility to label human pancreatic tissue by antibodies *ex vivo*, these studies are confined to relatively small tissue preparations [see e.g. (34, 41)]. Furthermore, clearing protocols, such as SHANEL (42), have been demonstrated to enable clearing of intact human organs (and the pig pancreas). Still the human pancreas presents significant challenges for antibody-based labeling procedures caused by insufficient reagent penetration and endogenous AF disturbances. The possibility to study any protein expression pattern throughout the human pancreas, in mm resolution, would have significant implications in this research field. It is likely that a holistic view of the human pancreatic anatomy, implementing high resolution optical 3D images, using specific molecular markers, will contribute to a greatly enhanced understanding of the pancreatic constitution. As previously exemplified in the mouse (8, 14, 18), it may not only contribute to unravel novel features of its normal anatomical organization. Such methodological advances may obviously also contribute to address a wide range of pathological/mechanistical features, on a molecular level, related to diabetes. For example, by offering the possibility to screen for and to perform “absolute” quantifications of e.g., islet mass distribution, immune-cell infiltration, amyloid deposition, endocrine cell maturity/function, or cellular ratios etc. Furthermore, they may provide a powerful approach to identify ROI´s or rare cell niches which would be much more challenging with the currently available technologies. Recently, Zhao et al. used purpose-built LSFM setups to demonstrate that this type of investigation would already be possible given that sufficient labeling could be acquired (42). Probably, future labeling protocols utilizing cameloid or shark nanobodies (43), which have much lower molecular weight as compared to common antibodies (approximately 12–15 *vs*. 150–160 kDa in size), will contribute to such developments. In view of the above, it is likely that advances in computational tools will be required for our possibilities to statistically assess and to visualize the gradually increasing data sets that are/will be possible to obtain by mesoscopic imaging techniques. Powerful computers running specific programs using algorithms for machine learning and/or deep learning may significantly contribute to facilitate analyses of complicated expression patterns and cellular distribution patterns in large organs preparations analyzed by high resolution OPT or LSFM. In this respect, these tools could enable greatly improved analyses of e.g., vascularization and innervation (44, 45), cellular responses, gene activation (46), automated and unbiased volumetric quantification of labeled features, such as islets or infiltrating immune-cells (47).

**Figure 2:**
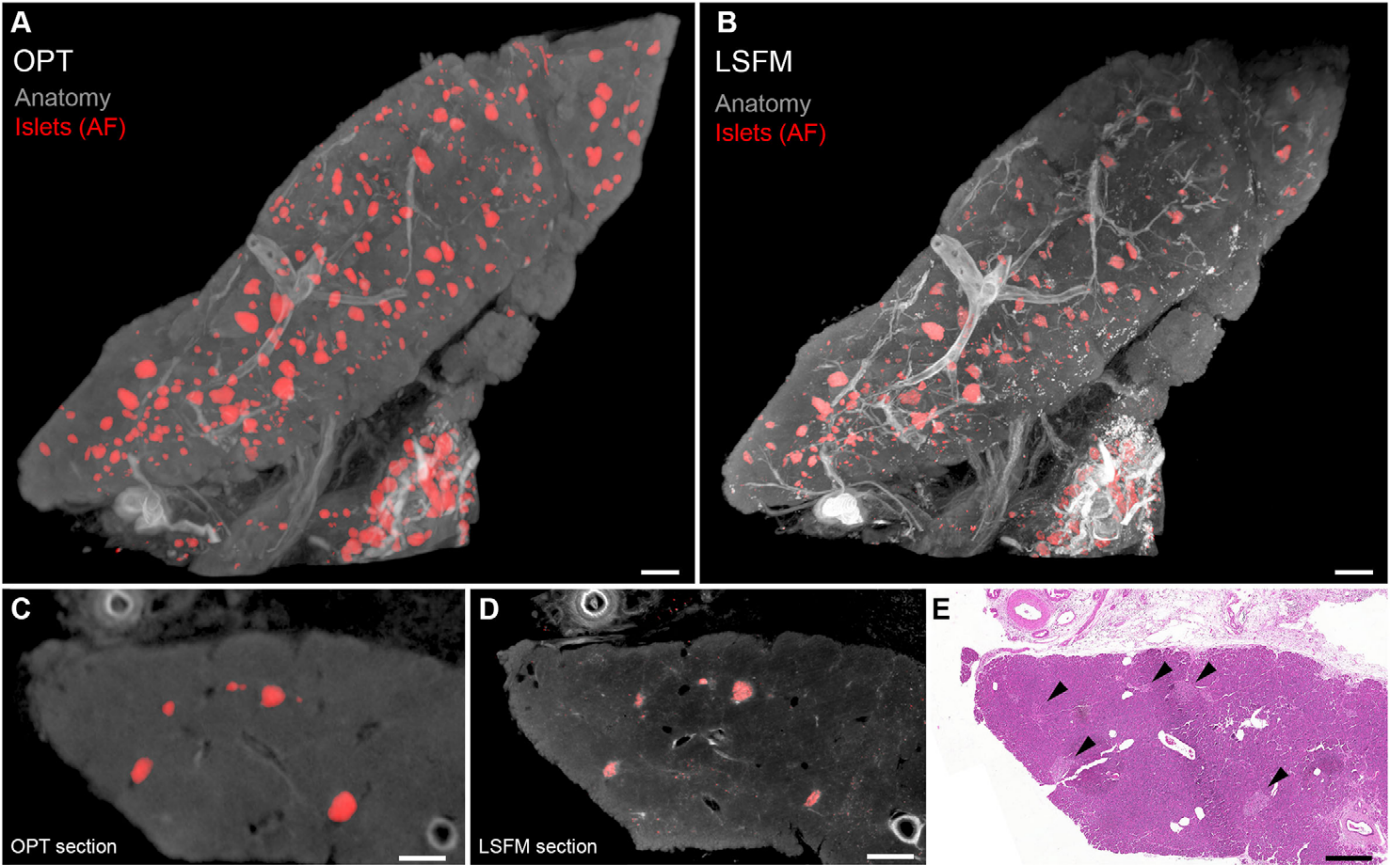
Optical projection tomography (OPT) and light sheet fluorescence microscopy (LSFM) imaging of human islets of Langerhans based on islet autofluorescence. **(A, B)** OPT **(A)** and LSFM **(B)** 3D reconstruction of human pancreatic tissue. The islets (red) are reconstructed based on their AF properties in the near infrared part of the spectrum and the “anatomy” and vessels (gray) in the visible part of the spectrum (GFP-channel). **(C, D)** Tomographic slices of the specimen seen in **(A, B)**. **(E)** Hematoxylin/Eosin staining of a tissue section obtained post-mesoscopic imaging corresponding to **(C, D)**. Islets are indicated by black arrowheads. As outlined in the text, OPT and LSFM are complementary techniques with pros and cons for different imaging scenarios, and most usually applied tissue processing protocols render them fully interchangeable. Data is obtained from Hahn et al. (48). Scale bar in **(A, B)** is 500µm and in **(C–E)** 400µm.

As mentioned before, the AF properties of the pancreas, and in particular the human pancreas, may significantly obstruct fluorescent 3D imaging assessments when using OPT and LSFM. However, the pancreatic AF properties differ significantly in distinct parts of the spectrum. By using narrow wavelength bands in a wide range of the spectrum (400–700nm), we could recently demonstrate that e.g., blood vessels, islets and even pancreatic malignancies could be visualized and segmented individually based solely on their AF properties both by OPT and LSFM (48) (see **Figure 2**). In this study, the far red to near infrared spectrum provided sufficient signal to noise ratio to enable quantitative 3D assessments of the islet mass distribution in >cm (3) sized pancreatic biopsies, based on the AF signal caused by the accumulation of, most likely, lipofuscin-like pigments in the islets. It should be noted however, that the accumulation of this lysosomal digestion product may differ between individuals of various ages and disease history. Therefore, specific molecular labeling would be advantageous for standardized assessments of islet mass.

Like SPECT and PET, OPT, and LSFM represent functional imaging techniques, designed to visualize specific cells or processes in a certain target tissue, normally using specific antibodies. The most important advantage of these functional techniques is that they exert great sensitivity and specificity for their target. On the other hand, however, they usually provide limited anatomical information. Although some anatomical reference information can be obtained from tissue autofluorescence in mesoscopic imaging, the anatomical detail remains limited for certain tissues. In nuclear images, this problem is often overcome by co-registering them with CT or MR images to create so called fusion images, providing an image that displays the specifically targeted signal onto the detailed anatomy of the tissue of interest. In theory, creating fusion images would also be possible for mesoscopic images of the pancreas, when co-registering them to *ex vivo* CT or MRI images of the target tissue, acquired after clearing. In the future, the possibility of creating mesoscopic fusion images might significantly contribute to the clinical significance of these imaging modalities in pancreatic disease since it would allow to visualize their specific high-resolution signal in its anatomical environment. Indeed, similar analyses with LSFM imaging as a functional modality have already been created for the brain (49).

## Concluding Remarks

During the past decade, mesoscopic imaging technologies have undergone dramatic developments. As such, they have opened the door for a wide range of highly specific molecular 3D analyses of the pancreatic anatomy and its constitution both in health and disease. Already now, they provide the possibility to perform holistic µm-resolution 3D imaging and accurate quantitative assessments of distinct cellular features of the rodent pancreas, a possibility that may be enabled in the human pancreas in the near future. It is our expectation that these rapidly evolving imaging technologies will soon contribute significantly to a better understanding of both the anatomical and molecular features of the normal and diseased pancreas as well as other tissues involved in endocrinological disorders.

## Author Contributions

TA, MH, SW, and UA wrote the manuscript. UA and MH made the figures. All authors contributed to the article and approved the submitted version.

## Funding

This work and the data from which figures 1 and 2 are derived was supported by the Swedish Research Council (grant no. 2017-01307), Barndiabetesfonden, the Diabetes Wellness Foundation (Sverige), Umeå University, The Novo Nordisk Foundation (grant no. NNF17OC0026794), the Juvenile Diabetes Fundation, the Kempe Foundations and the People Programme (Marie Curie Actions) of EU FP7/2007–2013/ under grant agreement no. 289932 by grants to UA.

## Conflict of Interest

The authors declare that the research was conducted in the absence of any commercial or financial relationships that could be construed as a potential conflict of interest.
